# Pre- and post-partum variation in wool cortisol and wool micron in Australian Merino ewe sheep (*Ovis aries*)

**DOI:** 10.7717/peerj.11288

**Published:** 2021-04-27

**Authors:** Gregory Sawyer, Dylan Russell Fox, Edward Narayan

**Affiliations:** 1 School of Science, University of Western Sydney, Penrith, Australia; 2 School of Life and Environmental Sciences, The University of Sydney, Sydney, Australia; 3 School of Agriculture and Food Sciences, Faculty of Science, The University of Queensland, St Lucia, Australia; 4 Queensland Alliance for Agriculture and Food Innovation, University of Queensland, St Lucia, Australia

**Keywords:** Wool microns, Fibre diameter, Stress, Pregnancy, Lambing, Wool cortisol, HPA-axis

## Abstract

An individual merino sheep’s output of wool production is influenced by synergistic interactions of sheep genetics, climate, farm management, and nutrition available to the whole flock. The price paid to the producer for this wool commodity is determined via numerous tested parameters and /or subjective appraisal of the raw greasy wool. This research investigated the level of variation in wool cortisol (a physiological stress biomarker) and wool micron (MIC) in Merino ewes (*Ovis aries*), pre-partum and post-lambing (lactation/lambs at foot), using maiden ewe (*n* = 38) managed in an outdoor paddock in a commercial farm. The key findings of this study are; (1) wool quality indicators showed a significant variation between pre- and post- parturition including significant reduction in MIC and (2) there was a negative correlation between wool cortisol levels and wool micron pre-parturition (rs = − 0.179, *p* < 0.05). This relationship between wool cortisol and wool micron was positive (rs = + 0.29, *p* < 0.05) during post-parturition suggesting that ewes with lambs at foot ended up with finer wool (reduction in fibre diameter) but they also maintained high levels of wool cortisol. Furthermore, the comfort factor, curvature, standard deviation and spin fineness of the wool were also significantly reduced post-parturition. The results of this study show that metabolic resources partitioning in ewe associated with pregnancy and lambing can result in a reduction in wool quality indices. The activity of the HPA-axis is attenuated during late gestation and parturition as a maternal adaptation; however, the results of our study show that wool cortisol remained similar between pre- and post- lambing. This result indicates that environmental stressors that may have been operating on farm (e.g., cold winter period) could influence on maternal physiological stress response however the exact level of influence of environment conditions on ewe stress levels and productivity traits (e.g., lambing success and wool quality) warrants further investigation. In conclusion, the use of top-knot wool sampling in combination with wool cortisol analysis provides researchers with a convenient method to quantify wool quality and physiological stress simultaneously under commercial sheep production.

## Introduction

Merino wool is highly sorted globally for the textile and fashion sectors. There is a great drive in the wool industry to continually improve research knowledge to bolster the efficiency of growing wool and wool quality as well (Rogers, 2006). Latest research shows that the growing wool on a live sheep and wool quality can be influenced by extrinsic factors such as climate, pregnancy status disease and nutrition (Gonzalez, Sacchero & Easdale, 2020; Sawyer, 2019).

### Raw wool characteristics

Raw (greasy–unprocessed) sheep wool is a complex structure that is made up of proteins known as keratin that grow from the blood-filled sac of the dermal papilla within the epidermis of the sheep (Lyne & Hollis, 1968). Within the raw wool fibre there are various components (in order) these are the cuticle (outside sheath), cortex, cortex cell, macrofibril, matrix, microfibril and the twisted molecular chain. The cortex is further broken down into paracortex and orthocortex. The raw wool cell structure can be influenced by in the animal’s interaction with three key factors. These factors (nutrition, disease and pregnancy) influence wool quality (fibre diameter, staple length, staple strength, yield, vegetable matter content position of break) (Cottle et al., 2013; Naidoo et al., 2016; Sawyer, Webster & Narayan, 2019; Ikusika et al., 2020) and wool production (kilograms produced) between shearing(s) (Narayan et al., 2020). Secondary influences include the age and sex of a sheep that affect the quality and quantity of the volume of wool being produced between shearing cycles (Cottle et al., 2013; Naidoo et al., 2016; Ikusika et al., 2020; Sawyer & Narayan, 2019). As the sheep ages, its early adult wool characteristics (micron, style, visual crimp) and wool quantity in grams diminishes. The exchanges of the primary and secondary factors within the animal’s life between shearing’s either promote or place limitations to processing ability of raw wool fibre.

### Wool production and stress interaction

Several previous studies that have highlighted the influence of stress on wool follicle development and growth, and wool quality indices using controlled experiments (see latest research in this field; (Weaver et al., 2020)). Raw wool fibres have also been identified as a reliable indicator of stress hormone (cortisol) levels at a point of time within the wool growth period (Sawyer & Narayan, 2019; Davenport et al., 2006; Nejad et al., 2014). There is an interconnection between quality of the wool fibre on the sheep’s body and the processing ability of the wool fibre that are determined by the animal’s reaction to its environment, not just its genetics. Research into how the pregnancy status of the ewe influences wool staple characteristics has been conducted for over two centuries from Darwin in 1859 (Darwin’s, 1859) (inheritance) through to recent research (Narayan et al., 2020; Sawyer & Narayan, 2019). Earlier research into pregnant ewes’ interaction and commodity output (wool growth - kilograms produced) decreases throughout gestation and even more reduced during the first month of lactation (Oddy, 1985). When the animal is stressed by its environment, the presence of high plasma cortisol levels can cause wool follicle shutdown (Sawyer & Narayan, 2019; Chapman & Bassett, 1970; Hansford & Kennedy, 1988) demonstrated within a housed sheep operation that pregnancy itself is a moderate stressor which caused fibre micron reduction in individually pen housed ewes. To our knowledge, this relationship has not been evaluated by testing the same wool sample for wool morphometrics and cortisol levels.

The glucocorticoid hormone cortisol, due to its multifaceted role in the physiological stress response, is the primary physiological biomarker for many observable phenotypic change in animals associated with activation of the hypothalamo-pituitary adrenal (HPA) axis (Yates, 2009). During the activation of the HPA-axis, cortisol is synthesised from cholesterol from the zona fasciculata of the adrenal cortex with stimulation by adrenocorticotropic hormone (ACTH) released from the anterior lobe of the pituitary gland, the pituitary is stimulated by corticotropic releasing hormone (CRH) and Arginine Vasopressin (AVP) secretagogues from the hypothalamus. Cortisol is the main glucocorticoid hormone in sheep (Fulkerson & Tang, 1979; Tilbrook & Clarke, 2006). Cortisol has a low molecular weight and lipophilic nature which attributes to its physiological (genomic and non-genomic) functions to regulate many cellular processes such as blood glucose regulation and immuno-suppression, and well-characterized regulation of the HPA axis on animal health and cognition events (Do, Eosu & Man-Ho, 2014; Fürtbauer, Solman & Fry, 2019). Non-invasive wool hormone methodology can be applied to determine longitudinal changes occurring in cortisol levels during pregnancy and post lambing (Sawyer, Webster & Narayan, 2019; Nejad et al., 2014). In wool, various hormones, including cortisol and progesterone, are captured in the wool structure from blood circulation to the capillaries of the dermal papilla, which is located in the individual wool follicles (Sawyer, Webster & Narayan, 2019; Do, Eosu & Man-Ho, 2014). The minimally-invasive wool collection method, undertaken during routine husbandry practices, can provide worthwhile biological information on the long-term changes in cortisol in response to environmental and management factors. This is due to the wool’s continual growth rate of slightly less than 1 cm/month depending on the strain of sheep breed. Furthermore, pasture quality or access to feed can also influence wool growth and quality as poor quality feed has been shown to reduce protein synthesis in merino sheep (Adams, Blache & Briegel, 2002; Ferguson et al., 2011).

### Raw wool processing

Raw wool fibre diameter is reported in the units of measurements of microns and measured as a millionth of a metre and presented as a mean average. Wool varies in microns from fibre to fibre, fleece to fleece, mob to mob and flock to flock. The visual changes in wool fibre can be seen mostly by the naked eye and it is current industry practice to analyse wool fibre diameter change along the wool staple *via* a laserscan (International Wool Textile Organization, Belgium).

The mean fibre diameter of the parcel of wool for sale is the most significant (70 to 80%) raw wool price determination (Schlink, 2009). When raw wool of the same micron is being purchased, there are other key factors that determine the resulting price paid by the buyer and received by the producer. These other wool quality determinates for wool processing are staple strength, staple length, vegetable matter base, processing yield and hauteur, the significance of each is determined by the individual wool processing requirements (Cottle et al., 2013; Ikusika et al., 2020). A staple of wool is a bundle of raw wool fibers approximately 5 to 10 mm collectively in thickness. The strength of the wool staple is a reflection of the force required to break the wool staple and is measured in newtons per kilotex (NKT). The NKT is then reported as a mean of the number of staples that have been assessed for staple strength. The lower the NKT, the weaker the wool staple when force is applied. Where the wool staple breaks under force is then weighed to determine the position of break (POB) in the staple. Due to various stressors (nutrition, pregnancy, disease, and environment interaction) the basic wool structure can alter from the desired phenotype to a thinning of the fibre thickness (fibre thickness reduction) (Ferguson et al., 2011).

The timing of this fibre thickness reduction in relation to the growth period of the overall length of the wool staple is measured as part of the standardized staple strength testing and is expressed as a percentage at the determined position of break (POB). The POB is located at either tip, middle or base category of the wool staple. It is widely accepted that the shorter length of the staple will have a greater strength than a longer staple due to the limited time influence of the environment to alter the fibre thickness. The mean fibre diameter, staple strength and POB do not affect the yield of the raw wool fibre. The yield is that amount of clean fibre content at a certain moisture content that is expected to be produced from a parcel of wool. It is expressed as a percentage through a mathematical equation of various components including percentages of dust, vegetable matter, residual grease and ash. A buyer of the raw wool determines maximum purchase price of a sale lot via the raw wool data made available including: micron, vegetable matter (VM) type, VM percentage and yield in the sale consignment.

Additional measurements (AM) of staple length, staple strength and position of break can only be used by the buyer if the wool staple is of combing length >45 mm in length and is predominately placed into worsted fabrics. If the wool staple is <45 mm in length, the buyer places their visual (non AM) assessment with that of the wools’ tested parameters (microns, VM, yield). These shorter wools have a different processing outcome known as carding wools and placed into woollen fabrics. Depending on the POB percentage in each category this may cause a possible reduction in wool buyer purchasing opportunity for the producer of the raw wool fibre. When the wool breaks in the middle of the staple, the resulting terminology is known as mid breaks and has an influence on the processing formulae known as TEAM. Wool purchasing can be affected by the expected processing TEAM formulae for predicting the length characteristics of combing wool. The TEAM formulae is calculated using all purchased lots in the processing consignment (Douglas & Couchman, 1997). TEAM is a widely accepted equation used to predict the Hauteur, CV Hauteur and Romaine in the processed wool top. Components of the TEAM formulae are: fibre diameter, staple length, staple strength, vegetable matter base, adjusted percentage of mid breaks and individual mill adjustment. A portion of wool processor output of quality and quantity is influenced by how the original sheep wool quality has been influenced by the sheep’s interaction to varying stressors within their environment between shearing’s.

The study did not intend to provide analysis of nutrition status, pregnancy output (singles, twins, triplets) or investigate other non-pregnancy influences on wool quality. It was hypothesised (1) that greater difference in wool fibre diameter will be experienced at lactation and (2) wool cortisol will be correlated negatively with wool microns pre-lambing suggesting that ewe with higher cortisol late gestation could result in finer wool fibre diameter post-lambing (thus accepting the traditional knowledge obtained by (Hansford & Kennedy, 1988) in an original pen based trial in 1988). We have also obtained data from previous commercial tested Merino wool from mid-side, pin bone and fleece to compare the variance of wool indices with the top knot wool samples used in our trial. Complete analysis of all price point determinants of the wool bale was not an objective of this study.

## Materials and Methods

All research samples were obtained during standard husbandry procedures by the Merino sheep owners. Western Sydney University (WSU) Animal Care and Ethics Committee (ACEC) approved this study (Protocol number A12345) and samples were analysed under the Biosafety and Radiation Safety Committee approval number (B12366) approved by the WSU Biosafety and Radiation Safety Committee (BRSC). All field and laboratory sampling methods followed exact protocols provided in our earlier research work on Merino sheep (Sawyer, Webster & Narayan, 2019).

### Animals and animal handling

The Merino sheep used in this study were all located at the property of Ooranook Pastoral Company, Braidwood, NSW, 2622 (GPS-35.251631, 149.867294). Maiden Merino ewes (*n* = 38) used for this study were randomly selected after health assessments of *n* = 70 naturally joined ewes (mated with rams) for 4 weeks, starting on the 26th of April, 2017. Condition of the ewes were assessed by the experimenter and any ewes with lack of condition were removed. However, body condition was not recorded due to equipment limitation. All sheep were shorn prior to the experiment to ensure that the wool samples were of similar length to assess wool cortisol over a standard period of time. The wool samples were collected at two different dates during summer of 2017, September (during very late stages of gestation–approximately two-four weeks prior to parturition) and December (ewes had given birth and ∼two-month-old lambs were at foot). The farm operator conducted shearing of the sheep as part of routine husbandry practice which allowed researchers to take a top knot (crown) wool sample from each ewe at the specified time period (Sawyer, 2019).

The sheep were run over one field (paddock) during the lifetime of the project. The field size is 462 acres (187 hectares), with 5 watering points. The feed on offer was native natural grasses local to the district and research fields with the naturally occurring grass being weeping grass (*Microlaena stipoides*). The field has 34% wooded timber for shelter from the environmental conditions (Sawyer, 2019).

The sheep were not fed any additional feed rations or supplementary diet substances during the research period. All sheep were drenched with Pyramid (av. liquid 10 mL) and DYNAMAX Controlled Release Capsules 3 times during the research period (January–post annual shearing, April–at time of ram introduction and September pre-lambing) (Sawyer, 2019). Pyrimide™ (Elanco) is a triple combination sheep drench containing 0.8 g/L abamectin, 25.5 g/L levamisole (as levamisole hydrochloride), 20 g/L albendazole, 0.4 g/L selenium (as sodium selenate) and 1.76 g/L cobalt (as cobalt EDTA). DYNAMAX (Boehringer Ingelheim) combination capsule drench with abamectin, albendazole, selenium (as selenium sodium EDTA), cobalt (as cobalt disodium EDTA)–no breakdown of each active ingredient is provided by manufacturer.

Each ewe was scanned using an ultrasound pregnancy scanner delivered by an authorized and trained animal pregnancy scanner to determine pregnancy status on the same day of the first top knot was removed in September.

### Wool sample collection

The wool sampling method is as exactly per our earlier research trial on Merino sheep (Sawyer, Webster & Narayan, 2019). Specifically, wool samples were collected during shearing events that occurred as part of the standard operations of Merino sheep farming. The topknot has not previously been used to individually measure wool fibre diameter. The topknot region was selected due to the novel testing for this new research and the expected application of this into future research. The top knot of the sheep is easily accessible when handling the sheep and allows for close to skin shearing without having to shear the whole animal which would not be suitable for all commercial sheep breeders. The purpose of using top knot wool was to demonstrate the potential application of this type of wool sample for testing cortisol and wool fibre diameter simultaneously in pregnant ewe. Please note, this does intend to replace any current industry practice however as a proof of concept pilot study to show that top knot provides a suitable wool sample for research purposes only. The same and complete area of the topknot was shorn throughout the trial. Wool was removed from the sheep by a specialist sheep shearer using an electric Heiniger shearing platform and shearing hand-piece (Shearing Supplies NSW PTY LTD, Australia). A portion of the top-knot wool sample that was collected from each ewe was submitted to Chad Wool for wool micron analysis. This portion was half of the full top knot collected and individual sample size was dependant on the size of topknot wool grown from each animal. A random sub-sample (50 mg) of wool staple was kept for wool cortisol analysis. Visual greasy raw wool characteristics including wool length, discoloration or grass vegetable matter content, was not recorded at time of collection. The wool samples for cortisol testing were wrapped inside aluminium foil and placed into a labelled Ziplock©bag and kept in a −80 °C freezer until assays (completed within 1 month of sample collection). Note:

### Wool cortisol enzyme-immunoassay

The wool cortisol assay method is exactly as per our earlier research trial on Merino sheep (Sawyer, Webster & Narayan, 2019). Wool samples analysis for cortisol is detailed in our earlier paper (Sawyer, Webster & Narayan, 2019; Sawyer & Narayan, 2019; Sawyer, 2019). Specifically, wool was washed in 90% isopropanol and air dried for 3 days using a dessicator prior to steroid extraction using 90% ethanol (all solvents were analytical grade). Wool cortisol was analysed using an enzyme-immunoassay using an anticortisol antiserum (R4866, supplied by the UC Davis Laboratory, California) diluted in ELISA coating buffer (Carbonate-Bicarbonate Buffer capsule (Sigma C-3041) and 100 mL Milli-Q water, pH 9.6), working dilution 1:15,000. This was followed by reactivity with Horseradish Peroxidase (HRP) conjugated cortisol label (CJM, UC Davies) diluted 1:80,000, and cortisol standards diluted serially (1.56–400 pg/well). Nunc Maxi-Sorp plates (96 wells) were coated with 50 µL cortisol antibody solution and incubated for a minimum of 12 h at 4 °C. Standards, including zeros and nsbs (non-specific binding wells), were prepared serially (2-fold) using 200 µL standard working stock and 200 µL assay buffer (39 mM NaH_2_PO_4_H_2_O, 61 mM NaHPO_4_, 15 mM NaCl). For all assays, 50 µL of standard and (1:10) diluted 90% ethanol extracted wool samples were added to each well, followed by 50 µL of the cortisol HRP. Each plate was loaded in under 10 min. Plates were covered with acetate plate sealer and incubated at room temperature for 2 h. After incubation, plates were washed 4 times using an automated plate washer (ELx50, BioTek) with phosphate-buffered saline solution (0.05% Tween 20) and then blotted on paper towel to remove any excess wash solution. Substrate buffer was prepared by combining 1 µL 30% H_2_O_2_, 75 µL 1% tetramethylbenzidine (TMB) and 7.425 µL 0.1M acetate citrate acid buffer, pH 6.0 per plate. The TMB substrate was added to each well that contained a standard sample at 50 µL to generate colour change. The plates were covered with an acetate plate sealer and left to incubate at room temperature for 15 min. The reaction was stopped with 50 µL of Stop solution (0.5MH_2_SO_4_ and Milli-Q water) added to all wells in the Nunc Maxi-Sorp plates. To determine hormone concentration in each sample plates were read at 450 nm (reference of 630 nm). Levels of wool cortisol were presented as ng/g dry wool weight.

### Wool laserscan assessment for experimental design

The wool sampling method is exactly as per the protocols available here (http://www.wooltesters.com.au/faqs). Specifically, snippets of raw wool (2 mm) were cut from samples of raw wool through a mini coring machine. The snippets were removed from the minicore and are suspended in a hexane solvent for a period of thirty seconds to wash and blend the fibre snippets together. A shot of compressed air was used to dry the snippets. A random sample was removed via tweezers from the washed and dried snippets and placed in to a dilute suspension in a mixture of isopropanol (propan-2-ol) and water (8% by volume). The suspension of snippets was transported through a measuring cell positioned in a beam of laser light. The reduction in intensity of the laser beam as the individual snippets passed through the beam of light, approximately 500 micrometres in diameter, was sensed by a detector and transformed, using a calibration look-up table, into a diameter in micrometres. The Laserscan assessment was conducted using an approved Laserscan machine by an independent wool company Chad Wool, Dubbo, New South Wales Australia.

### Statistics

Data were analysed using Excel. All data met the assumptions of data normality. Level of significance for all statistical analysis was *p* <  0.05. Definitions of wool traits assessed are as follows; **Fibre Diameter (MIC)**—the mean (average) micron result of a sample; **Standard Deviation (STDEV)**—measures in microns the spread of fibres either side of the mean FD in which 68% (two-thirds) of the fibres lie. The lower the SD the more uniform the wool; **Coefficient of Variation (COVAR)**—the SD divided by the mean FDx100. CV measures the FD variability as a percentage. CV tends to fall in the range of 15% to 30%, with 15% being very uniform wool, whilst 30% is highly variable wool; **Comfort Factor (COMFF)**—measures the percentage of fibres below 30.5 microns. Calculated as 100 minus the percentage of fibres below 30.5 microns; **Spinning Fineness (SPIN)**—calculated using the mean FD and CV to determine an “effective” fineness. This figure gives a measure of spinning performance. For wools of any particular micron the spinning performance decreases as the CV increases; **Curvature (CURV)**—measures how rapidly a fibre bends along an average standard short length of the fibre. Expressed as degrees of curve per millimetre of fibre (deg/mm). Curvature measurements usually range between 70 and 150 deg/mm. Curvature strongly relates to crimp frequency, therefore the higher the curvature the higher the crimp frequency.

We used paired t-tests to compare the level of significant difference in mean wool cortisol or wool indices between pre- and post- lambing periods. Furthermore, we conducted a Spearman (r) correlation to compare the wool cortisol and micron data separately for pre- and post- lambing periods.

Furthermore, we also tested the level of significant difference in wool micron taken using top-knot samples from our trial and sub-set of wool test data supplied by the Australian Wool Testing Authority (AWTA) for pin bone, mid-side and full fleece. This was to see whether the top-knot wool micron results were visually comparable to commercially available wool data.

Climatic data for the field site (Oornook, New South Wales, Australia) was obtained from the Bureau of Meteorology, Government of Australia (AGBo, 2016).

## Results

### Changes in wool profiles between pre- and post- lambing

The micron averages for Merino ewes for September 2017 assessment was 19.27 µm (*n* = 38). In the December 2017, assessment after ewes have lambed and had lambs at foot, the flock average micron was 18.44 µm (*n* = 38) a change reduction of 0.832 µm finer wool. Range: +1.2 microns stronger to −2.3 microns finer from the first assessment (September 2017) to second assessment (December 2017). This level of reduction in wool microns between pre- and post- lambing periods was significant (*p* < 0.05).

Through non-invasive assessment of wool, thirty-three of thirty-eight (84.6%) maiden Merino ewes recorded micron becoming finer up to 2.3 microns post-lambing. Six of the thirty-eight (15.4%) of the maiden Merino ewes assessed saw the microns becoming broader from pre lambing to post lambing.

Furthermore, wool parameters evaluated for pre- and post- lambing periods showed significant changes (see Table 1).

**Table 1:** Wool parameters in Merino ewe sheep. Wool parameter averages for *n* = 38 ewe sheep measured between pre- lambing and post- lambing. *P* value <0.05. MIC, fibre diameter (micron); STDEV, standard deviation; COVAR, covariance; COMFF, comfort factor; CURV, curvature; SPIN, spinning fineness.

Wool parameter	Average		*p* value
	2 weeks before lambing	Lambs at foot	
MIC	19.27	18.44	<0.05
STDEV	3.48	3.89	<0.05
COVAR	18.06	21.08	<0.05
COMFF	99.16	98.66	<0.05
CURV	95.39	86.74	<0.05
SPIN	18.34	19.77	<0.05

### Changes in wool cortisol between pre- and post- lambing

Mean (±S.E.M) WCM levels were 3.55 ±0.14 ng/g in September and 3.20 ±0.16 ng/g in December and not significantly different (*p* > 0.05).

**Figure 1:**
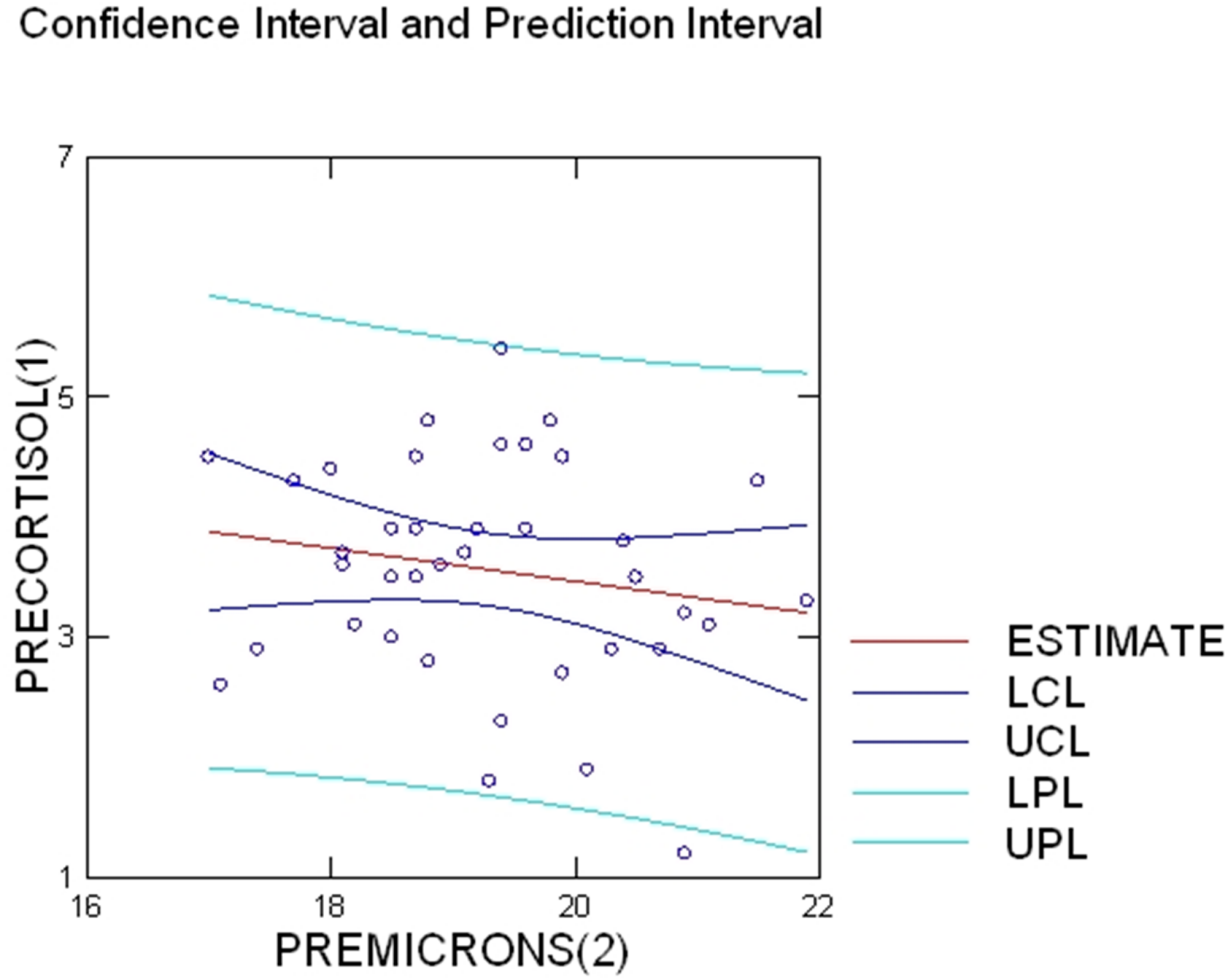
Pre-lambing wool micron and cortisol correlation. Shows the positive correlation between pre-lambing wool cortisol and wool microns as depicted by the upward progressing line of best-fit (*r* =  − 0.179). *R*^2^ and 95% confidence level has been shown (*R*^2^ = 0.03).

**Figure 2:**
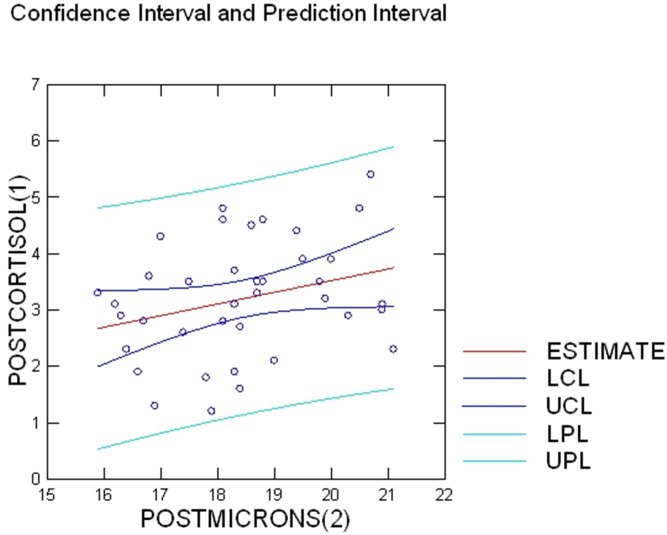
Post-lambing wool micron and cortisol correlation. Shows the significant negative correlation between pre-lambing wool cortisol and wool microns as depicted by the downward progressing line of best-fit (*r* =  + 0.29). *R*^2^ and 95% confidence level has been shown (*R*^2^ = 0.08).

Pre-lambing wool microns and wool cortisol levels produced a significant negative correlation (*r* =  − 0.179, *p* < 0.001); (Fig. 1). Post-lambing wool microns and wool cortisol levels produced a positive correlation which was also significant (*r* =  + 0.29, *p* < 0.001, Fig. 2).

### Variance in top knot wool micron compared to commercial sample types

See Table 2.

**Table 2:** Wool quality indices of wool sample types. The micron and covariance values for wool sample types including top-knot from our trial and other commercial sample types (pin bone, fleece and side sample).

	*n*	Min-Max	Average micron
Top Knot (current trial)	38	17–21.9	19.27
Pin Bone	50	16.9–23	19.56
Fleece	50	14.2–22.6	17.51
Side Sample	50	17.5–23.7	20

#### Climatic Data

Descriptive statistics is provided for climatic data for the year 2017 (Table 3).

**Table 3:** Field site climatic data. Descriptive Statistics of climatic data taken from the Bureau of Meteorology station in Oornook, New South Wales, Australia.

	T.Max (oC)	T.Min (oC)	Rain (mm)	Evap (mm)	Radn (MJ/m2)	VP (hPa)	RHmaxT (%)	RHminT (%)
Descriptive statistics								
Mean	17.23849558	3.885840708	1.149557522	2.492035398	14.5380531	9.213716814	46.46548673	94.71238938
Standard error	0.321376559	0.338135168	0.259827677	0.10312384	0.410816219	0.199972834	0.810459777	0.730529708
Median	16	3.55	0	2	12.9	8.2	44.8	100
Mode	11.7	1.6	0	1	11	7.1	49.8	100
Standard deviation	4.831349059	5.083286203	3.906066482	1.550291247	6.175921971	3.006250879	12.18388203	10.98226961
Sample variance	23.34193373	25.83979862	15.25735536	2.40340295	38.14201219	9.037544346	148.4469813	120.6102458
Kurtosis	−0.375564723	−0.738219159	19.13276081	−0.27966629	−0.604686511	0.513533915	1.569582805	6.777527004
Skewness	0.677539045	0.262160917	4.225731343	0.741427736	0.479548946	0.96629018	0.767656134	−2.56684888
Range	22.5	23.5	26.6	7.5	27.2	14.8	78.2	62.7
Minimum	9.1	−5.7	0	0.4	3.2	5	14.4	37.3
Maximum	31.6	17.8	26.6	7.9	30.4	19.8	92.6	100
Sum	3895.9	878.2	259.8	563.2	3285.6	2082.3	10501.2	21405
Count	226	226	226	226	226	226	226	226

## Discussion

In this study, firstly it was shown that wool microns (fibre diameter) in maiden Merino ewes reduced significantly between pre- and post-lambing periods with 84.6% of ewes expressing finer wool at lactation. Secondly, wool cortisol levels pre-lambing showed significant negative correlation with wool micron. The levels of wool cortisol and wool microns post- lambing periods were significantly positively correlated and is comparative to the previous housed ewe results in (Hansford & Kennedy, 1988). Of particular interest from this study is how the fining of the wool fibre as determined by changes in fibre diameter and how this may provide new understanding of the outcomes of the interaction of the animal in its environment and how the animal is managed for shearing pre and post lambing.

Of particular interest from this study is fineness of the wool fibre as determined by changes in fibre diameter from top knot wool samples taken between pre- and post-lambing. Secondly, wool cortisol from the top knot sample may also provide new understanding of the outcomes of the interaction of the ewe in its environment and how the animal can be better managed for shearing. As the ewe naturally becomes stressed during the time of lambing, it is important to know how much the fibre diameter reduces along the wool staple from sheep that reside within commercial sheep farming operations. Knowing this information prior to post-lambing shearing ewes post-lambing will assist the woolclasser in preparing the parcel of wool for sale by predetermining the expected tenderness of the wool staple and micron fineness when compared to other flocks on the same farm who have not experienced pregnancy stress (i.e., wethers castrated males wool). This will also influence which wools can be blended with other like wools prior to lotting the wools together for testing by the Australian Wool Testing Authority (AWTA). We acknowledge that other factors including disease and nutrition also affect wool diameter (Ward et al., 2016). This research was specific in wanting only to know how much the fibre diameter has reduced during lambing on a commercial farm so to compare the results of (Hansford & Kennedy, 1988). It was not designed to assess other influencing factors such as climate, nutrition, body condition score or disease. Furthermore, the use of the topknot for research opportunities to determine wool quality and stress hormone (cortisol) is unique and novel in its application. The use of the topknot for research purposes can provide further opportunities to examine other hormones in the wool through the collection of the wool samples at the top of the head also provides animal welfare benefits—not having to shear the whole fleece and workplace health and safety for wool collectors. A top-knot sampling method (as shown in Table 2 the wool indices measured in the top knot are comparable to other commercial sample types taken from Merino sheep), greasy raw wool fibre thickness (microns) was finer by up to 2.3 microns and on average 0.83 microns finer, which gave results similar to the earlier work undertaken in a controlled non-grazing trial by Hansford & Kennedy (1988).

Within the Australian Merino, various significant wool traits have changed including visual counts to measured micron, staple length growth *via* soft rolling skin development, nonetheless the impact of greasy raw wool fibre micron measurement reduction in pregnant ewes and in post pregnancy ewes is generally unchanged in thirty years of breeding when compared to the previous housed sheep study conducted by Hansford & Kennedy (1988).

Wool processing output is impacted by a variety of factors that are determined prior to the animal being shorn off its fleece. The ability for the producer to understand how much pregnancy stress contributes to wool fibre thinning due to micron change (up to 2.3 microns finer) may provide a better understanding on why shearing pre-lambing may benefit wool staple strength.

While it is stipulated that pregnancy and investment of body reserves into lactation as the major variables affecting wool microns. Other factors that are important include access to water, nutrition, handling, climate and predation may be playing a role (Nejad et al., 2014; Corner et al., 2010; Vaughan et al., 2016; Ward et al., 2016; Ralph & Tilbrook, 2016; Sawyer, 2019).

Previous research determined that gender and physiological status have an influence within the wool parameters of sheep (Cilek, Breeding & Medicine, 2015) however these areas were not considered in this specific focused research. The present study highlights the variation in wool fibre diameter and wool cortisol in maiden Merino ewes between periods of pre- and post- lambing. The findings also support the use of wool cortisol as a quantitative method for tracking physiological stress levels in sheep. Using a combination of top knot wool and wool cortisol tests, researchers can make reliable assessments of the physiological status and wool staple strength, and these data can be used to improve welfare and management practices (e.g., elimination of psychosocial and environmental stressors may lead to improved wool staple strength) (Sawyer, 2019).

During pregnancy all ewes were exposed to natural climatic variation on farm. Therefore, we believe that any significant difference in wool cortisol and wool microns should be attributed due to a combination of external (climatic and nutrition) and intrinsic maternal factors (e.g., investment in milk production and the stress associated with pregnancy). Certainly, environmental conditions could influence on the physiological stress levels of the ewes in gestation and post-lambing. We have demonstrated this correlation between physiological stress (indexed using faecal cortisol metabolites) and ewe performance (e.g., embryo quality of donor ewes) in more controlled AI/ET trials (Narayan, Sawyer & Parisella, 2018). The activation of the HPA-axis in animals operates along the allostasis threshold and the body maintains an internal balance of biochemical and physiological processes to maintain homeostasis or stability during crucial life-history stages such as reproduction. We believe that the levels of cortisol we have measured in wool during pre-lambing has captured the physiological response of the ewes towards maintenance of an internal balance while also supporting energetic demanding period of gestation through behaviours such as grazing and resting under the available environmental and landscape conditions. Furthermore, the post-lambing wool cortisol and wool micron has captured the physiological stress response of ewes to parturition and lactation associated stressors and feed on the ground (food availability) is also important. We hope that our data adds to the important discussion on ewe management and monitoring chronic stress using minimally invasive technology such as wool cortisol (Nejad et al., 2014; Weaver et al., 2020).

## Conclusion

This research provides a greater understanding that the initial hypothesis as determined in (Hansford & Kennedy, 1988) in trials undertaken twenty-nine years in housed sheep have a very similar fibre thinning outcomes to the genetically improved merino ewes of 2017. What was determined further was that as the raw wool fibre is produced via a blood sack and it acts as a “straw” that allows hormones to travel throughout the wool staple. How the animal interacts within its changing environment during pregnancy and during peak stressful periods including lambing will produce cortisol hormone that has an effect of fining the wool fibre by up to 2.3 microns on fibre diameter and overall have an effect on fibre quality. The producer may consider his time of shearing to reduce fibre breakage caused from lambing stress. Further research is required to best determine the ideal time to shear a sheep to optimize wool fibre production form pregnant ewes within various environment especially the rangelands and grasslands.

Furthermore it shows that the use of the topknot for wool research is a site that can provide a unique research capability to those researchers who are not confident in shearing a whole sheep and only want a small piece of fibre from the same sampling site time and time again. Finally the influence of the genetic makeup of the animal and whether the animal has a positive or negative interaction with the environmental factors, can cause the raw wool micron to increase or decrease (Narayan et al., 2020; Sawyer & Narayan, 2019; Chapman, 1989; Sawyer, 2019; Ferguson et al., 2011) and will need to be assessed with more research into sheep epigenetics as the environment changes.

##  Supplemental Information

10.7717/peerj.11288/supp-1 Supplemental Information 1 Field wool micron and cortisol data, and climatic data for 2017

## Additional Information and Declarations

### Field Study Permissions

The following information was supplied relating to field study approvals (i.e., approving body and any reference numbers): The owners of Oornook pastoral company (Mr. Gregory Sawyer) gave verbal approval for access to the sheep.

### Data Availability

The following information was supplied regarding data availability: The raw data measurements are available as a Supplementary File.
